# The Impact of Specific Viruses on Clinical Outcome in Children Presenting with Acute Heart Failure

**DOI:** 10.3390/ijms17040486

**Published:** 2016-04-01

**Authors:** Maria Giulia Gagliardi, Alessandra Fierabracci, Mara Pilati, Marcello Chinali, Carlo Bassano, Francesca Saura, Isabella Giovannoni, Paola Francalanci

**Affiliations:** 1 Medical and Surgical Department of Cardiology, Children’s Hospital Bambino Gesù, Piazza S. Onofrio 4, 00165 Rome, Italy; mara.pilati@opbg.net (M.P.); marcello.chinali@opbg.net (M.C.); 2 Immuno-Infectivology and Clinical Trials Area, Children’s Hospital Bambino Gesù, Viale S. Paolo 15, 00146 Rome, Italy; alessandra.fierabracci@opbg.net; 3 Operative Unit and Chair of Cardiac Surgery, Tor Vergata University, Viale Oxford 81, 00133 Rome, Italy; carlo.bassano@uniroma2.it; 4 Department of Laboratory Medicine, Children’s Hospital Bambino Gesù, Piazza S. Onofrio 4, 00165 Rome, Italy; francesca.saura@opbg.net; 5 Department of Pathology, Children’s Hospital Bambino Gesù, Piazza S. Onofrio 4, 00165 Rome, Italy; isabella.g@fastwebnet.it (I.G.); paola.francalanci@opbg.net (P.F.)

**Keywords:** myocarditis, cardiomyopathy, virus, echocardiography, biopsy, pediatric, longitudinal study

## Abstract

The presence and type of viral genomes have been suggested as the main etiology for inflammatory dilated cardiomyopathy. Information on the clinical implication of this finding in a large population of children is lacking. We evaluated the prevalence, type, and clinical impact of specific viral genomes in endomyocardial biopsies (EMB) collected between 2001 and 2013 among 63 children admitted to our hospital for acute heart failure (median age 2.8 years). Viral genome was searched by polymerase chain reaction (PCR). Patients underwent a complete two-dimensional echocardiographic examination at hospital admission and at discharge and were followed-up for 10 years. Twenty-seven adverse events (7 deaths and 20 cardiac transplantations) occurred during the follow-up. Viral genome was amplified in 19/63 biopsies (35%); PVB19 was the most commonly isolated virus. Presence of specific viral genome was associated with a significant recovery in ejection fraction, compared to patients without viral evidence (*p* < 0.05). In Cox-regression analysis, higher survival rate was related to virus-positive biopsies (*p* < 0.05). When comparing long-term prognosis among different viral groups, a trend towards better prognosis was observed in the presence of isolated Parvovirus B19 (PVB19) (*p* = 0.07). In our series, presence of a virus-positive EMB (mainly PVB19) was associated with improvement over time in cardiac function and better long-term prognosis.

## 1. Introduction

Acute myocarditis is a serious medical condition associated with high morbidity and mortality in both children and adults. Myocarditis may be initiated by a number of different environmental agents, such as viruses, bacteria, parasitic organisms, and drugs [1]. Dilated cardiomyopathy (DCM) can occur as a late sequela of acute or chronic myocarditis, either due to the persistence of the viral infection or to an autoimmune attack secondary to the exposure of an inciting virus [2,3,4].

Despite the recent promising results obtained from noninvasive diagnosis and prognostication of acute myocarditis (*i.e.*, MRI) [5], endomyocardial biopsy (EMB) remains an important diagnostic tool in children with unexplained DCM [6,7]. Inflammatory infiltrates within myocardial tissue samples are detected at histological examination and the identification of possible etiologic agents, such as cardiotropic viruses, can be obtained through polymerase chain reaction (PCR) [4,8,9,10], real-time PCR [11], reverse transcription PCR [12], or *in situ* hybridization [13]. Throughout the last 50–60 years, temporal fluctuations in the prevalence of different viruses associated with myocarditis have been documented [14]. In the last decades, Parvovirus B19 (PVB19) has become the more prominent virus amplified in EMBs of patients with myocarditis and DCM [15], both in adults [16,17,18] and children [19,20].

The aim of our study was to evaluate the presence of specific viruses in EMB specimens collected between 2001 and 2013 from children who were admitted to our hospital for acute heart failure and subsequently diagnosed with either myocarditis or DCM. In addition, we evaluated differences in clinical and echocardiographic features and long-term clinical outcome according to the presence and different types of viruses amplified in EMBs.

## 2. Results

### 2.1. Clinical Characteristics and Baseline Echocardiographic Findings

Clinical characteristics of the population are showed in Table 1. The age ranged from 3 months to 19 years with a median age at diagnosis of 2.8 years. Thirty-four children (54%) were female. According to the Dallas criteria [21], myocarditis (either acute lymphocytic, 22.5%, or borderline myocarditis, 14%), was diagnosed in 23 patients, while DCM was diagnosed in 40 (63.5%).

### 2.2. Polymerase Chain Reaction (PCR) Analysis

Viral genome was amplified in 19/63 cases (30%). In 17, a single virus was identified, while a combined infection was found in two. In particular, isolated PVB19 was detected in 17 EMBs (40.7%), EBV (Epstein–Barr virus) in two (25.6%), CMV (cytomegalovirus) in one (10.2%), HSV (Herpes Simplex) in one (2.5%). ENV (enteroviruses) and ADV (adenovirus) were never detected. In two cases, PVB19 was associated with HSV or EBV. We report the results of the PCR viral amplification in the three classified cardiac diseases (Table 2). Among the 19 patients in which PCR amplified viral genome, 12 had myocarditis (nine of these with acute myocarditis and three with borderline myocarditis), and seven had DCM. Of the 12 with myocarditis, 10 had detectable PVB19 (seven in the group with overt myocarditis, three in the group with borderline myocarditis), while two were positive for EBV (all in the group with myocarditis). In one case of myocarditis, a double infection with PVB19 and EBV was detected. PCR-amplified viral genome in 7 of the 40 children (17.5%) with DCM: five had detectable PVB19, one had CMV, and one showed a double infection with PVB19 and HSV.

### 2.3. Left Ventricular Volumes and Ejection Fraction

Mean left ventricular end-diastolic volume (EDV) at the time of diagnosis was 118.8 ± 60.5 mL/m^2^ with a mean ejection fraction (EF) of 30.8% ± 10.5%.

As shown in Table 3A, when dichotomizing the whole study population according to the presence or absence of viral genome, we found that mean indexed EDV was 93 ± 29 mL/m^2^ in children with virus, and 103 ± 38 mL/m^2^ in children without virus (*p* < 0.05). Similarly, baseline EF was 36% ± 10% in the children with virus, and 26% ± 0% in children without virus (*p* < 0.01). At the time of discharge, a larger improvement in EF was found in patients with virus-positive EMB (+18%) as compared to those with virus-negative EMB (+14%; *p* < 0.05).

As shown in Table 3B, comparing patient groups according to the histology pattern, mean indexed EDV was 98.7 ± 42 mL/m^2^ in children with myocarditis, 106.7 ± 23 mL/m^2^ in children with borderline myocarditis, and 134.9 ± 79 mL/m^2^ in children with DCM (*p* value for trend in one-way ANOVA = 0.039). EF was 30.9% ± 10% in the children with myocarditis, 31.1% ± 14% in those with borderline myocarditis, and 30.7% ± 10% in patients with DCM (*p* = Not Significant).

At the time of discharge, a significant increase in left ventricular EF could be observed in all study sample groups, with a markedly higher improvement in patients with myocarditis (+22%) and borderline myocarditis (+18%) as compared to +12% in those with DCM (*p* for both *vs*. DCM <0.01).

In *post-hoc* analysis, the two patient groups (myocarditis + borderline myocarditis *vs*. DCM) were further sub-divided according to the absence or presence of viral-genome-positive PCR in order to define four subgroups: DCM-negative PCR (*n* = 33), DCM-positive PCR (*n* = 7), myocarditis (MYO)-negative PCR (*n* = 11), and MYO-positive PCR (*n* = 12). When comparing echocardiographic data of these groups, only a minor effect of positive PCR on left ventricular (LV) diastolic volumes could be observed (probably also due to the small number of observations), but a significant difference was found in EF recovery. In details, from admission to discharge, while in the DCM group the presence of positive PCR was associated with a lower increase of EF (+6.5%) compared to DCM with negative PCR (+14%, *p* = NS (not significant)), in the myocarditis group, the presence of positive viruses was related to a significant recovery in EF (+25%) as compared to patients with myocarditis and negative viral presence (+15%, *p* < 0.05).

### 2.4. Follow-up and Prognosis

**Figure 1:**
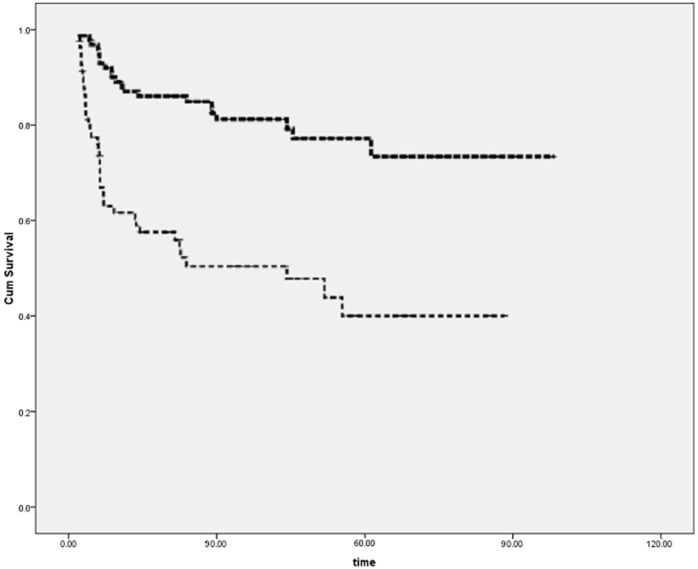
Kaplan Meyer Survival curve according to endomyocardial biopsies (EMB) (virus positive EMB in thick dashed line and virus-negative EMB in thin dashed line) (*p* < 0.001, see text for explanation).

During clinical follow-up (mean 4.2 years, range 6–120 months), 27 events occurred, including 7 cardiac deaths and 20 cardiac transplants. The incidence of adverse cardiac events was significantly lower in the group of patients with either clear-cut or borderline myocarditis (26%) as compared to the group without myocarditis (63%; Figure 1; *p* < 0.001). When dichotomizing our population on the basis of viral presence, virus-positive PCR was associated with a significantly higher rate of freedom from events as compared to virus-negative PCR patients (10.5% *vs*. 57%, *p* < 0.001).

**Figure 2:**
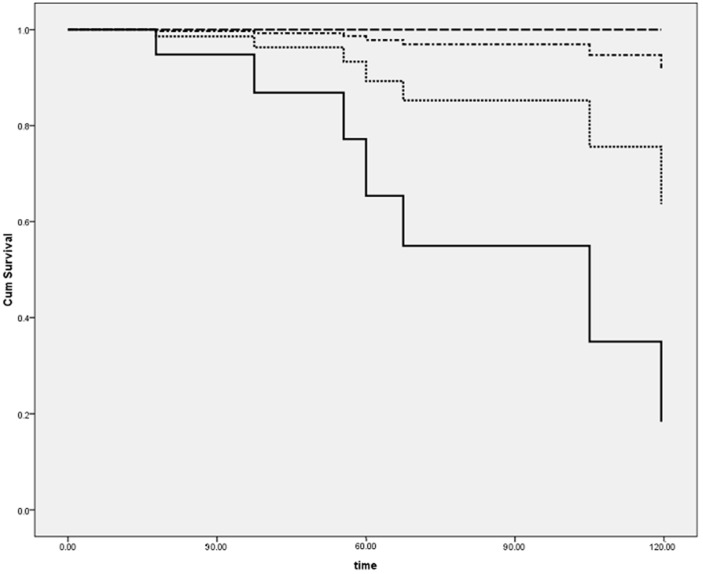
Analysis performed by separating groups according to the diagnosis (dilated cardiomyopathy (DCM) *vs*. myocarditis) and presence of a positive EMB. Consequently, four subgroups were created: DCM-negative polymerase chain reaction (PCR) (continuous line), DCM-positive PCR (dotted line), myocarditis (MYO)-negative PCR (dotted-dashed line), and MYO-positive PCR (dashed line). See text for explanation.

When further analyzing the population as four separate groups on the basis of diagnosis and detection of viral genome as previously described, virus presence was associated with a markedly better outcome both in patients with myocarditis (8.3% *vs*. 45.5%, *p* < 0.01, Figure 2; *p* < 0.01), and in the DCM group (14.3% *vs*. 60.6%; *p* < 0.01).

In Cox regression analysis, after correction for differences among groups in age, gender, and EF improvement over time, both the presence of myocarditis (HR (Hazard Ratio): 0.23; 95% CI: 0.12–0.42) and of a virus-positive EMB (HR: 0.52; 95% CI: 0.25–0.79) were independent predictors of a better outcome (both *p* < 0.05).

There were no significant differences in event rate when separating patients with virus-positive EMB according to differences in virus type (*i.e.*, PVB, EBV, CMV), possibly also due to the low incidence of events in the virus-positive EMB group.

## 3. Discussion

The aim of the present study was to identify the prevalence and type of a specific group of viruses, as well as the clinical outcome in children affected by dilated cardiomyopathy or myocarditis. It is well known that the spectrum of presentation of paediatric myocarditis ranges from minor flu-like illness with chest pain to acute cardiogenic shock in a previously healthy child. Although myocarditis has a high recovery rate, fulminant myocarditis as well as intractable dilated cardiomyopathy require aggressive supportive care, including mechanical circulatory support, as a bridge to transplantation or to recovery [22,23]. Heart transplantation remains the only final therapeutic option for children with dilated cardiomyopathy or myocarditis with intractable severe heart failure [24,25].

In our study sample of 63 young patients, a negative outcome was observed in 43% of the population (including 7 cardiovascular related deaths and 20 children requiring heart transplant). As expected, a worse outcome was observed in children with DCM; however, in regression analysis we found that the presence or absence of a virus-positive EMB was also an independent predictor of events. Presence of specific viruses assessed by PCR was detected in approximately 30% of children. Viral presence was associated with a significant improvement during the hospitalization time in cardiac function and in a significantly higher survival rate at follow-up in the whole study sample, both in myocarditis and in DCM. It should be noted that while in the absence of virus on EMB, incidence of events was 57%, in the presence of virus-positive EMB, only two events were recorded (10.5%; *p* < 0.05). We need to point out, however, that the main limitation of our study is that other viruses (like HSV6) or bacteria (*i.e.*, mycoplasma) known to be involved in myocarditis were not tested and could theoretically be involved in causing worse prognosis.

Some authors have already suggested a predominant role of PVB19 in the etiology of myocarditis and DCM [26,27]. However, PVB19 infection is relatively common in humans, with 50% of the population having detectable IgG antibodies by the age of 15, increasing to 90% in the elderly [28,29]. In addition, the mechanisms through which PVB19 induces inflammation of the myocardium are not yet well recognised, although it has been suggested that PVB19 could play a role in the induction of endothelial dysfunction and coronary artery vasospasm [30,31,32]. Towbin *et al.* [19,33] suggested that evidence of PVB19 infection could be observed in young patients with myocarditis and postoperative paediatric cardiac transplant patients presenting unexplained rejection.

Our study demonstrates that the effect of viral infection (most commonly a PVB19 infection) is characterized by a “transient” cardiac dysfunction, possibly secondary to the effect of the aforementioned endothelial dysfunction caused acutely by the virus. This dysfunction is often reversible, and the presence of viral genome appears to be associated with a more favorable long-term prognosis.

Our study shows that over one third of our patients experienced a negative clinical outcome at follow-up (death or transplant), and young patients with myocarditis, either overt or borderline, tend to recover more often than those with idiopathic DCM.

Considering the limitation underlined above for our study regarding the specific group of viruses analysed, viral etiology was not common in our clinical setting, as it could be identified in only approximately 30% of the study sample. However, viral presence was associated with a significant early improvement in cardiac function and better long-term prognosis, with a detectable independent effect, regardless of whether the viral genome was observed in children with a diagnosis of myocarditis or DCM.

In conclusion, our findings suggest that performing EMB with PCR analysis in paediatric patients presenting with acute heart failure—not sustained by either structural cardiac anomalies, neuromuscular diseases, or systemic metabolic syndromes—provides useful prognostic information. The presence of specific viruses was indeed detected in only 30% of patients, PVB19 being the most commonly isolated virus. Better prognosis was associated with the presence of viral etiology, although the main limitation of our study is that important viruses or bacteria were not tested. These could theoretically be involved in causing a worse prognosis.

## 4. Materials and Methods

### 4.1. Study Population

Between January 2001 and February 2013, 63 patients, from newborn to teenagers, were referred to our hospital for acute heart failure (Table 1). Inclusion criteria were: initial presenting symptoms of acute cardiovascular collapse or acute heart failure, such as new-onset dyspnea with associated hepatosplenomegaly, physical exam findings of reduced cardiac output, or evidence of dilated heart on chest X-ray and echocardiograms. Children with less-severe symptoms were admitted to the cardiology ward and treated conventionally, while patients with severe heart failure were directed to the intensive care unit and received intravenous treatment. After routine evaluation, all children underwent an echocardiographic study. Echocardiographic criteria used in the diagnosis of myocarditis or cardiomyopathy included ventricular dilatation and reduced left ventricular ejection fraction, in the absence of cardiac structural anomalies, anomalous origin of left coronary artery, systemic skeletal muscle disease, and/or valvular disease or arrhythmias. All patients underwent heart catheterization. Informed consent was obtained from all patients and parents or legal guardians. The study was approved by the local Institutional Review Board (IRB) of Bambino Gesù Children’s Hospital, in November 2000 (cod. 199602R016), regulating the use of human samples for clinical studies, that are collected for routine investigations. Furthermore, the IRB approved the informed consent procedure.

### 4.2. Follow-up

After hospital discharge, patients were followed clinically for up to 10 years (mean follow-up 4.2 years; range 6 months to 10 years) at 6-month intervals, with physical examination and echocardiography if clinically necessary. Cardiac related death or transplant were considered as primary endpoints of the study.

### 4.3. EMB Histology

EMBs were obtained from the right side of the septum in each patient by means of a flexible 6 French bioptome using transjugular or femoral vein approach. Seven or eight EMB specimens were obtained from each child, frozen in liquid nitrogen and stored at −80 °C for subsequent extraction of RNA and DNA for PCR analysis. According to the “Dallas criteria” [21], myocarditis was diagnosed when there was histological evidence of inflammatory infiltrates within the myocardium associated with myocyte degeneration and necrosis of non-ischemic origin. By immunohistochemistry, abnormal inflammatory infiltrate was defined as follows: ≥14 leucocytes/mm^2^, including up to 4 monocytes/mm^2^ with the presence of CD3-positive T lymphocytes ≥7 cells/mm^2^. We recognized three types of cardiac histological patterns: acute lymphocytic myocarditis, borderline myocarditis, or no myocarditis.

### 4.4. Detection of Viral Genomes

We searched for the following DNA viruses: ADV, CMV, HSV1/HSV2, EBV, and PVB19, using pre-synthesized primers, based on published sequences or purchased from Amplimedical SpA, Bioline Division (Turin, Italy). RNA of enteroviruses (ENV) (Amplimedical, 5’ UTR region Poliovirus 1–3, Coxsackie virus A1–A22 and A24, Coxsackie virus B1–B6, Echovirus 1–9, 11–21, 24–27, 29–33, ENV 68–71) were also searched.

### 4.5. Statistical Analysis

Results were expressed as mean ± SD. All variables were tested for normality using the Kolmogorov–Smirnov test. Data among groups were compared by Student *t*-test for continuous variables and by *Chi*-square test (with Monte Carlo method for computation of exact 2-tailed *p* value). Analysis of variance (ANOVA) and analysis of covariance (ANCOVA) were performed to analyze differences among two or more groups, when correcting for relevant confounders. Paired *t*-test for continuous variables and *Chi*-square with McNemar’s test for categorical variables was used to compare repeated measures performed at admission and discharge. The Kaplan–Mayer curves with product limit estimator was used to calculate freedom from mortality and/or heart transplantation between different groups. Multivariate Cox regression analysis was used to compute hazard ratios in order to identify predictors of clinical outcome, independently of significant clinical and echocardiographic confounders. A *p* value below 0.05 was considered statistically significant.

## Figures and Tables

**Table 1:** Characteristics of the population studied.

Characteristics	Value
Patients	63
Female	34 (54%)
Median age at diagnosis (range)	2.8 (0.1–19.5)
Fever *	13
Symptoms at presentation	≤7 days
EF at diagnosis	30.8% ± 10.5%
Myocarditis	22.5%
DCM	63.5%
Mean follow-up ±SD	4.2 ± 5
Deaths	14 (7.4%)
Heart transplantation	39 (20.6%)

DCM: dilated cardiomyopathy; EF: ejection fraction; Symptoms: Tachycardia, Dyspnea. * All patients presenting with fever suffered from myocarditis.

**Table 2:** Polymerase chain reaction (PCR) results: viral DNA genome amplified.

Diagnosis	EMBs	Viral Genome	
		PCR Positive	PCR Amplifier
Myocarditis	14	9	PVB19 (*n* = 7) EBV (*n* = 2) PVB + EBV (*n* = 1)
Borderline myocarditis	9	3	PVB19 (*n* = 3)
DCM	40	7	PVB19 (*n* = 5) CMV (*n* = 1) PVB + HSV (*n* = 1)

EMB: Endomyocardial biopsy; PVB19: Parvovirus B19; EBV: Epstein–Barr virus Virus; CMV: Cytomegalovirus; HSV: Herpes Simplex Virus.

**Table:** (**A**)

Variable	Viral Genome (+)	Viral Genome (−)	*p* Value
LV-EDV (mL/m^2^)	93 ± 29	103 ± 38	<0.05
Baseline LV-EF (%)	36 ± 10	26 ± 10	<0.01
EF change at follow up (%)	+18	+14	<0.05

LV = left ventricular; EDV = end-diastolic volume; EF = ejection fraction.

**Table:** (**B**)

Variable	Myocarditis	Borderline Myo	DCM	*p* Value for Trend
LV-EDV (mL/m^2^)	98.7 ± 42	106.7 ± 23	134.9 ± 79	<0.05
LV-EF (%)	30.9 ± 10	31.1 ± 14	30.7 ± 10	NS
EF change at follow up (%)	+22	+18	+12	<0.05

LV = left ventricular; EDV = end-diastolic volume; EF = ejection fraction; Myo = myocarditis; NS: Not Significant.

## Abbreviations

EMB	Endomyocardial biopsy
PCR	Polymerase chain reaction
PVB19	Parvovirus B19
DCM	Dilated cardiomyopathy
MRI	Magnetic resonance imaging
EF	Ejection fraction
EBV	Epstein–Barr virus
CMV	Cytomegalovirus
HSV	Herpes simplex virus
ENV	Enterovirus
ADV	Adenovirus
n	Number
EDV	End-diastolic volume
NS	Not significant
Myo	Myocarditis
LV	Left ventricular volume
DNA	Deoxyribonucleic acid
RNA	Ribonucleic acid
